# Human milk: insights on cell composition, organoids and emerging applications

**DOI:** 10.1038/s41390-025-04458-3

**Published:** 2025-10-02

**Authors:** Misba Majood, Rajini Rao

**Affiliations:** Department of Physiology, Pharmacology and Therapeutics, The Johns Hopkins University School of Medicine, Baltimore, MD USA

## Abstract

**Abstract:**

Human milk is far more than a source of infant nutrition. It is a dynamic, living fluid packed with cells, bioactive molecules, and a complex microbiome that shapes neonatal development and lifelong health. Recent advances have illuminated the remarkable cellular diversity of human milk, including epithelial, immune, microbial and stem cells, each contributing essential biological functions. Milk contains distinct membrane-bound structures in the form of milk fat globules and extracellular vesicles that package a diverse cargo of lipids, proteins and nucleic acids for neonate nutrition, development and immune regulation. This review explores the composition of human milk, highlighting its nutrient and bioactive components and discussing growing concerns of xenobiotic and viral burden. We describe how milk-derived cells offer non-invasive windows into lactation biology and how emerging 3D mammary organoid models, particularly those generated from human milk cells, provide unprecedented tools to study breast development, lactation disorders, and regenerative therapies. We outline the potential of milk cells and extracellular vesicles in neonatal care, personalized medicine, and biobanking, while addressing current technical challenges and future research opportunities. By harnessing the unique properties of human milk, we stand at the threshold of transformative insights into maternal-infant health and novel biomedical applications.

**Impact:**

- Up to date summary of bioactives, living cells and membrane bound compartments found in human milk.
- Primer on human mammary organoid technology, including advantages, recent advances and step by step methods.
- Highlights the unrealized potential of human milk in organoid technology, therapeutics, and regenerative medicine.

## Introduction

Human milk has evolved to be the optimum infant food.^1,2^ Produced by the mammary glands, milk provides nutrition, immune protection, and developmental support to the neonate. Milk is a complex fluid that consists of myriad components including nutrients (lipids, proteins, carbohydrates), bioactive molecules (vitamins and immunomodulatory factors), various cells (lactocytes, macrophages, stem cells and bacteria) and cell-like components (milk fat globules and extracellular vesicles) with important biological functions.^3^ Together, the nutrient and non-nutrient components of human milk are highly effective for the survival and healthy development of human infants. However, nutritional deficiencies in human milk (e.g., pre-term milk^4^), coupled with the increasing burden of xenobiotics,^5^ pose significant and long-term health risks to the infant. A contemporary understanding of human milk composition and the state-of-the-art tools and technologies applicable to milk research is prerequisite to improving the quality and safety of human milk.

Breastfeeding is a key public health strategy with major health organizations recommending exclusive breastfeeding in the first six months of life.^6,7^ However, infants are frequently weaned before this period due to insufficiencies in lactation, as reported by 40–50% of breast feeding persons in the US and 60–90% world-wide.^7^ Examination of gene and protein expression in lactating breast tissue could serve to gauge the differentiation and functionality of breast tissue during lactation and to evaluate the potential for successful lactation. This could be especially beneficial for addressing lactation challenges, including issues with milk supply and delayed secretory activation in lactating persons with premature infants. However, studies on lactation historically rely heavily on animal models which fail to fully recapitulate human lactation physiology, or on healthy breast tissue that is difficult to obtain, especially during lactation, and has limited scalability. Differences between human and animal models include the number and location of mammary glands, the timing and trajectory of mammary gland development, and the composition of milk.^8,9^

In contrast, as we describe in this review, human milk is a readily available and non-invasive source of milk fat globules, extracellular vesicles, microbiota and living cells from the lactating breast that could offer valuable insight into the natural function of this organ and its associated disorders. Recent advancements in stem cell technology and 3D cell cultures may be applied to human milk cells, opening new avenues for studying lactation biology. We describe how organoids could lead to a deeper understanding of the human mammary gland, and we speculate on the potential use of milk-derived stem cells in regenerative medicine. Finally, we explore the potential biotechnological and clinical applications of human milk.

## Cell-derived components of human milk

**Fig. 1:**
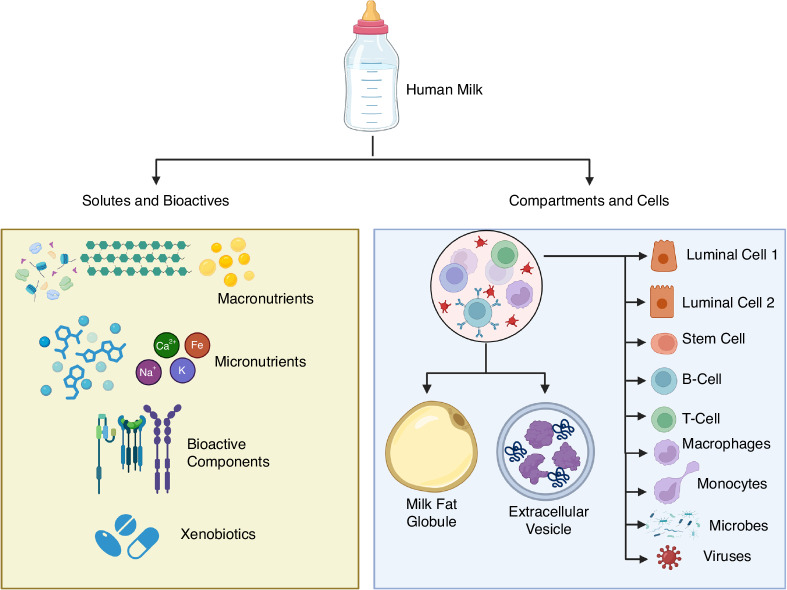
Diverse components of human milk. This figure depicts the multifaceted nature of human milk as both a nutritional and immunological source for infant development. Left panel: biochemical components include macronutrients (carbohydrates, proteins, and lipids), micronutrients (essential minerals like calcium, iron, sodium, and potassium), bioactive components (such as immunoglobulins, hormones, and growth factors), and xenobiotics (external compounds like medications and environmental contaminants). Right panel: cellular fraction features milk fat globules for lipid transport, extracellular vesicles carrying proteins and RNA, and various cell types, including luminal epithelial cells, stem cells, immune cells (B-cells, T-cells, macrophages, and monocytes), as well as microbes and viruses that contribute to gut colonization and immune modulation in infants.

Human milk is a complex fluid composed of diverse solutes, membrane bound structures and living cells^3,10,11^ (Fig. 1). The composition of milk varies with the stages of lactation, extent of nursing, maternal genetics and diet, health of the breast feeding dyad, whether the infant is born full-term or preterm, and many other factors.^4,12^ Establishing a thorough and reliable reference that encompasses all essential nutrients and bioactive factors in human milk will require standardized population studies in the future. A comprehensive understanding of the components of human milk could inform on lifestyle and diet choices that impact milk production and composition, and better formulation of supplements for the changing needs of infant nutrition in the first few years of life.

### Macromolecules and micronutrients

The solutes in milk include macronutrients, micronutrients, bioactive components including immunomodulatory and growth factors, and a variable composition of xenobiotics. On average, milk comprises ~87% water, 7% lactose, 3.8% fat, and 1% protein, with lactose being a predominant carbohydrate averaging 62 g/L.^11^ The fat content is primarily composed of triglycerides, polyunsaturated fatty acids, cholesterol, and phospholipids that are critical for cognitive and brain development. Whey proteins (e.g., lactoferrin, lysozyme, secretory IgA) and casein support growth, intestinal health, and immunity.^3,13^ Human milk oligosaccharides constitute the third most abundant class of solutes, with more than 150 distinct structures influenced by maternal genetics.^14^ They serve as food substrate for gut bacteria and when fermented, release beneficial compounds that improve intestinal barrier integrity, and modulate immune function.^15^ Other bioactive components like beta-casomorphins enhance gut function and regulate satiety.^16,17^ Examples of micronutrients are vitamins and minerals, including ionic and complexed calcium, magnesium, iron, zinc, and copper, that are critical to meet infant needs.^18^ Human milk contains numerous immunomodulatory factors and growth-promoting elements that extend its benefits beyond nutrition. Immunoglobulins, predominantly secretory IgA, provide passive immunity by safeguarding mucosal surfaces and protecting against infections during the early months when the infant immune system is still developing.^10^ Growth factors such as Epidermal Growth Factor (EGF), Insulin-like Growth Factor-1 (IGF-I), and Vascular Endothelial Growth Factor (VEGF) are critical for intestinal development, vascularization, and tissue repair, especially in preterm infants.^19^ Adiponectin, a hormone found in human milk, regulates infant metabolism and is inversely correlated with infant Body Mass Index (BMI), suggesting a potential role in preventing childhood obesity.^20^ Collectively, these milk components support physical growth, cognitive development, and immune health of the neonate, underscoring their value during infancy.

### Milk fat globule

When observed under a microscope, the visual characteristics of human milk are remarkable. Despite being a fluid, human milk possesses significant structure in the form of compartments that sequester nutrients and bioactive compounds. An exemplary illustration of this arrangement is the milk-fat globule, a natural colloidal assembly ranging in diameter from 0.1 to 20 μm composed of a triglyceride core encapsulated within a complex membrane derived from the secretory mammary epithelial cell.^21^ The lipid core is surrounded by a tripartite membrane: an inner monolayer of proteins and polar lipids with the fatty acid tails facing the globule core that is derived from the cytoplasmic leaflet of the endoplasmic reticulum and a bilayer of polar lipids, proteins, glycoproteins and cholesterol derived from the plasma membrane during the budding process. In addition, there is an entrained layer of cytoplasm about 10–20 nm wide between the inner single membrane and outer bilayer.^22,23^

The milk-fat globule sequesters specific proteins, growth hormones, and vitamins, which may be incorporated into the limiting membrane.^21^ The membrane functions as a stabilizing barrier between the aqueous milk components and segregated fat globule and facilitates the regulated liberation of lipolysis products and the transfer of polar substances into the aqueous phase of milk serum. In addition to it nutritional value, the human milk fat globule plays a major role in protecting the infant against infection through the lipase dependent production of free fatty acids and monoglycerides that exert potent antimicrobial and antiviral properties in the infant gut, reviewed in ref. ^24^ Mucins, lactadherins and other glycoproteins in the milk fat globule membrane bind to pathogens, preventing their attachment to the gastric mucosa.^24^

### Extracellular vesicles

Exosomes and extracellular vesicles (EV) constitute another important cell-derived component of milk.^25^ They vary widely in size depending on their mode of production.^26,27^ Fusion of multivesicular endosomes with the plasma membrane in mammary epithelial cells gives rise to 30–100 nm exosomes. Direct budding of vesicles from the plasma membrane in a process called ectocytosis leads to the formation of microvesicles, with a wider diameter range of 100–1000 nm. In addition, extracellular compartments can arise from apoptotic bodies and have annexin V as a marker. These nanoscale vesicles encapsulate different bioactive molecules, including microRNA (miRNA), protein, and lipid, that survive the acidic milieu of the neonate intestine and may be endocytosed by intestinal cells to exert immunomodulatory and regenerative effects.^28^ The cargo content of EVs in transitional milk was found to depend on gestational age, with differences observed between preterm and term milk.^29^ As discussed ahead, the important role of extracellular vesicles in signal transduction, delivery of bioactive cargo, and maternal-infant communication is increasingly recognized.^30,31^

### Viruses

Human milk can be a reservoir for certain viruses and serve as a transmissible route for infant infection. For this to happen, the virus must infect and replicate in mammary epithelia and be secreted into milk. Furthermore, for infection to be successful, the virus must survive contact with the oral and digestive tract of the infant, proliferate and exit from enterocytes, and enter the bloodstream for systemic infection.^32^ Studies in the 1980’s established that human milk contains both cell-free and cell-associated particles of human immunodeficiency virus type 1 (HIV-1) that can be transmitted to breast fed infants. Paradoxically, milk from HIV-positive people has potent HIV-inhibitory activity and breast-fed infants have a lower rate of acquiring AIDS.^33^ During the COVID pandemic, concerns that lactating persons could transmit SARS-CoV-2 through milk combined with shortage of formula-based alternatives led to extensive testing and evaluation, with the conclusion that SARS-CoV-2 infection through human milk has limited transmission risk, and benefits for infants far outweigh the risk.^32^ Since 2024, surprisingly high titers of the highly pathogenic avian influenza virus HPAI H5N1 have been found in raw dairy milk, underscoring the importance of pasteurization and potential for human transmission, including to lactating persons.^34^ Although influenza is considered a respiratory virus, HPAI H5N1 virus shows a marked tropism to the mammary gland,^35^ which is the primary replication site in dairy cows leading to mastitis and high viral loads in milk.^36^ The unexpected ability of HPAI to replicate efficiently in the mammary gland has been linked to sialic acid receptors: recent immunohistochemistry reports showing expression of specific sialic acid receptors essential for HPAI binding in human mammary tissues^37,38^ highlights the risk of human transmission which could be a rising concern for lactating persons.

### Xenobiotics

Although human milk is the gold standard for infant health and nutrition, it may contain varying levels of xenobiotics, including pesticides and industrial contaminants, mycotoxins, heavy metals and pharmaceuticals. These foreign compounds occur as environmental contaminants, medications, or are found in cosmetics and food, entering the maternal bloodstream through absorption, inhalation or ingestion.^39^ By partitioning into milk fat by maternal transfer, or transported across the mammary epithelium by efflux transporters such as the breast cancer resistance protein BCRP,^40^ xenobiotics are passed on to the infant where they exert harmful effects on infant development, act as endocrine disrupters, neurotoxins or allergens with long lasting health repercussions. Most biomonitoring studies use targeted analysis and can miss emerging or unknown xenobiotics, whereas non-targeted methodologies and multi-omics approaches, using gas chromatography and mass spectrometry for example, can identify novel compounds in human milk.^41^ Other challenges include developing guidelines for acceptable daily intake and evaluating aggregated and cumulative exposure from multiple sources.

## Cellular diversity of human milk

Human milk includes diverse populations of maternal-derived epithelial, immunological, microbial and stem cells. The number of cells varies with lactation stage, with peak levels reported in colostrum (146,000 cells per milliliter), and decreasing postpartum.^42^ As discussed below, bulk and single cell RNA sequencing studies have demonstrated that the cellular composition of milk changes throughout lactation. Gene expression cluster analysis on longitudinal samples obtained from human donors revealed up to ten broad cell types, including seven types of immune cells and three epithelial cell compartments.^43^ These studies reveal the existence of distinct mammary cells and their patterns of gene expression in human milk, emphasizing the intricate and biologically diverse cellular characteristics of human milk.

### Epithelial cells

The presence of epithelial cells in human milk was initially believed to be a byproduct of apoptosis. However, the discovery that many of these cells are viable and able to form mammospheres in primary cultures suggests that these are cells that detach from the ducts and alveoli during milk synthesis and secretion. There is evidence that milk cells delivered to the infant subsequently infiltrate into different organs for the benefit of the offspring.^44,45^

Epithelial cells, identified by expression of the mature mammary epithelial cell markers cytokeratin 18 (CK18) and CK19,^46^ are the most abundant cells of the mammary gland and critical for maintaining the structure and function of the organ.^47^ There are two categories of epithelial cells: ductal epithelial cells, which line the mammary gland ducts and alveolar or luminal epithelial cells, which differentiate into lactocytes during in milk production.^48^ One study showed that lactocyte epithelial cells accounted for an average of 81.7+/− 24% of all cells per milk sample, across both donor and lactation stage.^43^ It is unclear if basal/myoepithelial cells are in human milk: CK14 was detected in milk cells, but it is a marker common to both mammary progenitor cells and myoepithelial cells.^49^

All epithelial cells found in milk express genes involved in milk synthesis. However, subsets of these cells are differentially enriched for secretory gene expression (“secretory lactocytes”), tight junction function, cell cycle regulators or cytoskeleton functions pointing to their origin within discrete subdomains of structure or function within the lactating mammary gland.^43,50^ Whereas all lactocyte epithelial cells expressed lactalbumin (*LALBA*), Claudin 4 (*CLDN4*) and Krueppel-like factor 6 (*KLF6*)) highlighted the so-called LC1 cluster whereas xanthine dehydrogenase (*XDH*), casein alpha S1 (*CSN1S1*) identified a larger group of LC2 cells.^51^ These studies and many others reveal that the expression of different markers in human milk cells tracks with their stage of differentiation. A complex transcriptional program controls biosynthesis of milk and response to signals throughout lactation. Both the diversity and abundance of epithelial cells increased over the course of lactation, although cycling and secretory lactocytes decreased in proportion, possibly as an outcome of increased specialization over time.

### Immune cells

Macrophages and lymphocytes found in human milk migrate from the blood stream and maternal mucosal immune system, particularly the gut and respiratory tract, and are drawn to the mammary gland by specific signaling molecules and adhesion molecules.^52,53^ The presence of immune cells in human milk varies depending on the stage of lactation and the health of both the birthing parent and infant.^54^ During the initial stages of lactation, as in colostrum, a considerable proportion of human milk cells is made up of leukocytes, including macrophages, neutrophils, and lymphocytes. Macrophages comprise the most abundant immune cell type, at 50.5+/− 34% per sample.^52^ These cells travel through the intestinal mucosa and enter the infant’s bloodstream to strengthen immune defense and development.^55,56^ Flow cytometry analysis of human milk cells revealed an infiltration of CD45^+^ cells in response to mastitis and other infections. The correlation between milk immune cell counts and mastitis could be harnessed to develop diagnostic test for the disease which is a major cause of unrecoverable milk production.^57^ Intriguingly, there are hints of shifting macrophage composition in transcriptional metadata encompassing infant health, feed supplementation and daycare attendance, although larger and more robust datasets will be needed to tease out correlations.^43^

### Stem cells

Early reports on the identification of a population of nestin and p63 positive cells in human milk,^58,59^ together with demonstrations that cells obtained from human milk could expand, differentiate and propagate in culture pointed to the presence of a resident population of stem cells.^60,61^ Stem cells in human milk express pluripotency markers including OCT4, SOX2, and NANOG which comprise the critical transcription factor circuitry found in human embryonic stem cells.^62^ These cells are scarce in the non-lactating breast but are activated during pregnancy to direct the development of mammary tissue to a lactating organ. Milk stem cells can develop into a wide range of cell types, encompassing those from all three germ layers (endoderm, mesoderm, and ectoderm), a hallmark of pluripotency.^63^ In contrast to embryonic stem cells, milk stem cells did not form tumors when injected subcutaneously into immunocompromised mice in the teratoma assay.^62^ While the relative abundance of stem cells in human milk is unclear, they are ingested in significant quantities by the nursing infant.

Similar to milk leukocytes, stem cells ingested through milk may diapedese through the intestinal epithelium and enter the blood circulation of the infant to be distributed to various niches in the body. This could result in tissue microchimerism, continuing a process that has been observed in utero.^64^ Strikingly, studies in mouse models have revealed the integration of milk-derived stem cells into brains of suckling pups.^65^ The observation that maternal transplants are better tolerated in individuals who have been breast fed suggests that infiltrating stem cells from human milk may confer immune tolerance to maternal antigens in the infant, strengthening the case for human milk over formula milk.^66^

### Microorganisms

Human milk contains over 200 microbial phylotypes. In terms of abundance, about half can be assigned to a common milk microbiome and the remaining show individual variation.^67^ Initially, microbial presence in milk was attributed to contamination from the infant’s oral cavity or maternal skin, or from maternal infection. These mechanisms, however, do not explain the presence of microorganisms in precolostrum secreted prior to parturition,^68^ or the detection of anaerobic species found in the gut.^69^ There is emerging evidence for an entero-mammary pathway by which bacteria found in maternal gut are sampled by dendritic cells that can reach through tight junctions in the epithelial gastrointestinal lining.^57^ These bacteria travel through the lymphatic system to reach the lactating mammary gland. In human trials of women who received oral administration of lactobacilli strains, the presence of these bacteria in the milk was subsequently detected in >50% of participants.^70,71^

Infants can greatly benefit from the wide variety of probiotics found in human milk. Among the microbial variety, *Bifidobacterium* and *Lactobacillus* stand out as particularly noteworthy. *Bifidobacterium* is essential for maintaining a well-balanced gut microbiota, boosting the immune system, and safeguarding against infections.^71^ These bacteria play a crucial role in the digestion of human milk oligosaccharides, supporting the growth of other beneficial microbes and preventing proliferation of harmful pathogens.^72^ *Lactobacillu*s, a crucial microbial species, plays a vital role in preserving infant gut health. It achieves this by producing lactic acid, which effectively reduces the pH levels in the intestines and inhibits the growth of detrimental bacteria.^73–75^ There are several other noteworthy microorganisms worth mentioning. One of them is *Akkermansia muciniphila*, which plays a crucial role in fortifying the intestinal barrier and mitigating inflammation.^76^ Additionally, human milk contains *Staphylococcus* and *Streptococcus*, which have significant impact on the infant’s developing immune system, bolstering its effectiveness and safeguarding against disease.^77^

Metabolites generated by milk microorganisms, including short-chain fatty acids such as acetate, butyrate and propionate, have crucial functions in maintaining gut health, regulating the immune system, and supporting energy metabolism. These metabolites play an important role in promoting infant immune tolerance, mitigating inflammation, and maintaining gut health.^78,79^ Indeed, human milk is the exclusive source of short-chain fatty acids in the first year of life before the maturation of the infant microbiome. The protective role of maternal milk is especially critical in preterm infants whose reduced gut microbial diversity increases their susceptibility to pathogenic bacteria.^80^ Overall, microorganisms and their byproducts in human milk have a probiotic effect, promoting the growth and well-being of babies by strengthening the immune system, promoting a healthy gut, and safeguarding against various infections.^81^

## Emerging applications of milk

Bioactive compounds and cells isolated from milk are useful in both basic and disease-oriented applications. Milk-derived cells may be used in vitro studies to model mammary gland biology and lactation, and in understanding breast cancer, mastitis, and other lactation-related conditions. Potential therapeutic applications include the use of milk-derived stem cells for tissue engineering and regenerative therapies. The immunological properties of milk cells could be harnessed for developing new immunotherapeutic strategies. Here, we summarize the tools and techniques in applications of milk cells.

### Therapeutics

#### Drug delivery

Milk exosomes have many features conducive to their application in drug delivery, including stability, protection of cargo, oral bioavailability, scalability, low immunotoxicity, and ability to cross the intestinal barrier.^82^ Milk extracellular vesicles may be loaded with drugs, vaccines or nucleic acids for therapeutic and gene editing applications. Studies indicate that human and bovine milk vesicles can traverse the whole gut, remaining intact against enzymatic degradation in the acidic environment of the stomach, thereby, improving the delivery of their cargo to target cells.^30,83^ These advantages suggest that milk-derived exosomes could be modified to deliver therapeutic agents to diseased tissues, although the full potential of this technology remains to be realized. Developing robust and scalable procedures for the production, and refining strategies for precision and safety will be key to successful use of this platform.

#### Regenerative medicine

Stem cells obtained from human milk are ethically acceptable, pluripotent and non-teratogenic.^60,62^ In this respect, milk stem cells resemble umbilical cord and bone marrow-derived pluripotent cells that also display lower tumorigenic potential in immunodeficient mice where they can integrate into damaged tissue. Markers of neurons (β-tubulin, nestin and MAP2) as well as glia (s100b) have been detected in milk stem cell-derived lineage, making them potential candidates for cell replacement therapy in neurodegenerative disorders.^84^ Human milk derived spheroids cultured in appropriate differentiation media have the capacity to secrete milk protein (mammospheres), insulin (pancreatic beta-cell organoids) or express troponin T (cardiospheres).^62^ Interestingly, the tolerance of human milk-fed infants to maternal cells could be considered a form of “natural stem cell therapy” and bodes well for their application in regenerative medicine.^60^

#### Immunomodulation

Human milk has antibacterial and immunomodulating properties, and has traditionally been used in many cultures for the treatment of skin problems like dermatitis and eczema.^85^ More specifically, extracellular vesicles derived from human milk are increasingly recognized as important mediators for intestinal health by protecting against inflammation, promoting epithelial regeneration and modulating the composition of gut microbiota. Exosomes purified from both raw and pasteurized human milk, as well as from colostrum or mature milk, were effective in protecting against necrotizing enterocolitis in mouse models.^86–88^ MicroRNA cargo in EVs were found to shield neonates against inflammatory diseases, including necrotizing enterocolitis, through their regulation of immune signaling.^29^ Notably, human milk EV-associated miR-29 induced activation of intestinal stem cells to promote epithelial proliferation and repair damaged mucosa.^89,90^ The ability of milk EVs to modulate various inflammatory pathways such as the TLR4-NF-κB signaling cascade and NLRP3 inflammasome activation demonstrates their potential as therapeutics for inflammatory bowel disease (IBD) and necrotizing enterocolitis (NEC).^91^ At physiological concentrations, EVs were found to increase the barrier function of infant oral mucosa by targeting multiple signaling pathways.^92^ In the same way, EVs seemingly bolster the neonatal intestinal barrier, affecting mucus layer formation and modulation of tight junction proteins, with enormous implications for conditions associated with increased intestinal permeability, such as allergies and autoimmune disease.^93,94^

### Diagnostic medicine

#### Diagnostic applications

Beyond its nutritional value, human milk offers enormous diagnostic potential for insights into maternal and infant health, from the detection of infectious agents to early warning signs for infants at risk for neurodevelopmental disorders and microbial analysis for childhood asthma and atopy.^95^ Using milk as a tissue-specific biospecimen, one study identified significant differences in DNA methylation in subjects with subsequent diagnosis of breast cancer.^96^ Studies on human milk antibodies to respiratory pathogens have evaluated the effect of infection and maternal vaccination.^97^

#### Pharmacogenetics

Human milk is a source of DNA and can be analyzed in pharmacogenetic studies, helping to understand how individuals respond to medications. Maternal genetic factors can significantly affect milk pharmacokinetics and infant exposure to medication. Thus, pharmacogenetic differences in membrane transporter expression and/or activity impact the concentrations of key nutrients and drugs in human milk.^40^ For example, a single polymorphism in the *ABCG2* gene encoding the BCRP transporter alters milk levels of nifedipine in hypertensive lactating persons treated with this drug.^98^

### Biomanufacturing

#### Bioactives

Beyond infant nutrition, bioactives in milk have a range of potential applications in food industry, pharmaceuticals and nutraceuticals. These include the biomanufacturing of human milk oligosaccharides as probiotics, by large scale production using either cell- based approached, transgenic plants or microbial fermentation.^99,100^

#### Artificial or lab-grown milk

There is public interest in harvesting milk-like fluids produced from cultured lactogenic human mammary cells for a variety of ethical and practical reasons.^101,102^ Lab grown milk could be more sustainable than traditional dairy, reduce greenhouse gas emissions and use less land and water resources. Unlike lab-grown meat, milk-producing cells themselves are not harvested which means that they may be re-used to continuously generate milk and may be genetically engineered for quantity and quality without the milk itself being genetically modified. To produce raw milk from bioreactors, mammary epithelial cells are cultured on straw-like capillary devices either in the inside-out or physiologically correct topology to secrete their product into the surrounding medium or into the capillary lumen, respectively.^103^ Although promising, these efforts are still in early stages and face uphill challenges in economics of scale and limitations in reproducing many critical components of human milk such as immune cells.

### Research and development

#### Biobanking

Traditionally, milk banks have collected, screened, stored and distributed milk to infants needing access to the nutritional and immunological benefits of human milk. Milk biobanks also serve as valuable storage facilities for research and clinical purposes.^104^ Donor milk offers an abundant source of milk components including lactocytes, milk fat globules and extracellular vesicles that could provide insight on transcriptome and proteome changes through the course of lactation. For example, donor milk obtained from milk banks was an effective source of extracellular vesicles used to demonstrate protection against inflammation in enteroids and necrotizing enterocolitis in mouse models.^86^ Cell biobanking specifically refers to the methodical gathering, handling, and preservation of human milk cells from nursing women, typically using non-invasive techniques. The process entails the isolation of the cells, evaluation of their quality, and the subsequent preparation for cryopreservation.^105^ Optimal treatment and storage conditions are essential to prevent cellular deterioration and guarantee their viability for future scientific investigation. Pasteurization should be avoided to preserve cell components. These cells can be utilized by researchers to examine the impact of milk on newborn growth, immunological function, and disease prevention.^106^ Compared to the banking of umbilical cord derived stem cells, human milk derived stem cells have the potential to be collected in greater numbers and banked for future applications.^107^ Thus, from a clinical perspective, milk cells possess significant potential for the advancement of infant care and individualized therapy.

#### Generation of mammary organoids

Mammary organoids are three dimensional structures that recapitulate the complexity of the mammary gland, offering insights into lactation, mammary development and disease states including breast cancer.^108^ Mammary organoids are typically obtained from breast tissue. However, there is increasing interest in deriving mammary organoids from viable epithelial cells isolated from human milk, although published studies are scarce. We consider the advantages, applications and limitations of mammary organoids in greater detail below.

## Mammary organoids

Since 1987, 3D culture systems have been established to generate organoids that replicate a wide range of organs. These organoids comprise diverse cell types and exhibit self-organization akin to their in vivo behavior through processes of proliferation and differentiation. Consequently, they establish structures that preserve the original organ identity in vivo.^109^

### Advantages of mammary organoids

The use of in vitro organoid models for lactation and mammary research avoids the ethical, practical and cost issues associated with animal and human models. Furthermore, organoids generated from human cells more accurately replicate human physiology in comparison to animal models. Organoids can also be cultivated in a controlled biomimetic environment, allowing for systematic investigation of experimental and biological variables. The use of genetic tools coupled with real time imaging allows lineage tracing during organoid development. Organoid models are increasingly used for investigating diseases associated with lactation, including breast cancer, and for evaluating personalized therapeutic interventions within a system that more closely resembles individual human physiology.^110^

### Advances in mammary organoid research

In recent years, lab-grown organoids have replicated the intricate structures of mammary organoids in a controlled environment, offering valuable perspectives on biological processes and disease mechanisms. Primary mammary organoids derived from mouse tissue have significantly contributed to our understanding of the mechanisms behind mammary branching morphogenesis, particularly regarding the roles of the extracellular matrix and stromal cells within 3D culture models.^111,112^ Tumor organoids have been used for drug screening^113^ and a biobank with over 100 primary and metastatic breast cancer has been established^114^ with significant potential to advance personalized medicine, enabling dosage determination tailored to the specific needs of each patient. The incorporation of accessible and numerically controlled bioprinting systems has facilitated accurate spatial organization and scalable generation of consistent human mammary organoids, addressing the constraints of arbitrary cell distribution observed in conventional 3D culture techniques.^115^ These advancements enhance experimental consistency and throughput while facilitating the creation of customizable organoid arrays to examine intricate cell-cell and cell-matrix interactions pertinent to mammary gland biology and diseases.^116^

### Sources for generation of lactating mammary organoids

Human-specific organoids have been generated from breast tissue obtained from reduction mammoplasty, prophylactic mastectomy, breast biopsy, or resected cancerous tissues from donors or patients. Rosenbluth et al. successfully produced mammary organoids that exhibited the expression of both luminal and basal cell markers (CK8 and CK14, respectively).^117^ These organoids exhibited ductal and alveolar morphologies that closely resembled those present in the native mammary gland. Key breast tissue markers^118^ including estrogen receptor, progesterone receptor, and HER2neu, were preserved within the developed organoid model.

Induced pluripotent stem cells (iPSCs) are somatic cells that have undergone reprogramming, enabling them to differentiate into various cell types, encompassing all endodermal, mesodermal, and ectodermal lineages. Mammary-like organoids derived from human iPSC (hiPSC) display appropriate gene and protein expression and could be induced with lactogenic hormones to express milk protein.^119^ Human iPSCs can be derived from cells specific to individual patients, offering enhanced opportunities for personalized disease modeling and therapeutic approaches. This method mitigates many ethical issues typically linked to alternative sources such as tissue or embryonic stem cells.

Stem cells from human milk have self-renewal and pluripotency characteristics, which make them a promising source for generating mammary organoids. Although there have been reports of culturing human milk cells as spheroids,^62^ there are few published protocols showing differentiated mammary organoids derived from milk, likely due to the small population of viable stem cells in human milk. The yield of viable epithelial cells per mL of human milk varies widely with biology (lactation stage, time since expression, parity, donor health), sample features (foremilk vs hindmilk, fat content, transport/holding conditions), isolation settings, and downstream factors that together determine organoid-forming efficiency. As such, generalizable volume to yield conversions are not yet available. Nevertheless, we believe human milk has the potential to be a useful and physiologically relevant source for lactating mammary organoids in the future. Future studies, with larger sample sets and harmonized protocols, should focus on systematically reporting milk volume processed, cell yield per mL, viability, epithelial enrichment, and organoid-forming efficiency, which will ultimately allow the field to establish practical volume guidelines.

As enumerated in Table 1, there are distinct advantages and disadvantages associated with each of these sources for mammary organoid generation. Breast tissue is a source of differentiated mammary cells that readily assemble to natural tissue architectures. However, tissue collection requires invasive procedures, with significant limitations in scalability and issues of donor variability. These disadvantages are obviated by iPSC-derived organoids, but they are expensive and time consuming to generate. Milk-derived cells offer an excellent compromise as a non-invasive and readily available source of lactating cells and, as such, hold great promise for mammary organoid formation.

**Table 1:** Comparison of mammary organoids derived from mammoplasty tissue, induced pluripotent stem cells (iPSCs), and human milk based on key properties.^61,114,127–130^

Properties	Mammoplasty tissue	Induced pluripotent stem cells (ipsc)	Human milk
Stemness	Multipotent	Pluripotent	Multipotent
Teratoma formation	No risk	High risk	No risk
Differentiation	Already differentiated	Needs multiple growth factors and differentiation protocols	Already differentiated
Collection	Invasive (requires surgery)	Non-invasive (requires reprogramming of somatic cells)	Non-invasive (milk is collected from lactating women)
Production cost	Moderate to high cost	Very expensive	Low to moderate cost
Long-term propagation	Difficult, as they are primary cells	Easy, stable lines may be established	Difficult, as they are primary cells
Cell isolation	Easy, via digestion or mincing method	Reprogramming is challenging	Difficult, due to low viability and cell count
Variation	Donor dependent variation	Low variation once cell line is established	Donor and batch dependent variation
Breast physiology	Mimics breast tissue closely	Close, but not 100% (requires precise differentiation cues)	Mimics lactating cell physiology
Cell availability	Limited to tissue donors	Unlimited with proper culture methods	Limited by availability and lactation stage
Ethical considerations	Involves surgical procedures, raising ethical concerns	Ethical concerns related to reprogramming and genetic modifications	Few ethical concerns because of non-invasive collection
Application in research	Suitable for direct mammary studies and personalized medication	Highly versatile for modeling and regenerative studies	Useful for studying lactation specific pathways

### Generation of lactating mammary organoids

**Fig. 2:**
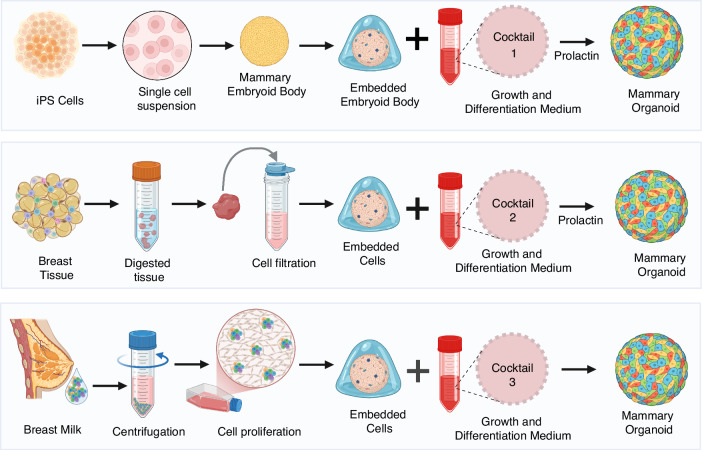
Mammary organoid generation. Step-by-step process for generating mammary organoids from three different sources: induced pluripotent stem cells (iPSCs), breast tissue, and milk-derived cells. Each approach follows a distinct isolation and differentiation protocol using key growth factors and signaling molecules to promote lineage specification. *Cocktail 1* contains parathyroid hormone for mammary lineage differentiation; hydrocortisone, insulin, FGF10, hepatocyte growth factor for branch and alveolar formation, and hydrocortisone, insulin, FBS for lactation. *Cocktail 2* contains R-spondin, neuregulin, FGF7, FGF10, EGF, noggin, A83-01, Y27632, SB202190, B27, NAC, nicotinamide, and *Cocktail 3* contains R-spondin, neuregulin, FGF7, FGF10, EGF, noggin, A83-01, Y27632, SB202190, B27, NAC, nicotinamide. Milk-derived organoids do not need prolactin as they are already lactogenic. The final organoids, cultured in a supportive extracellular matrix, exhibit heterogeneous cellular composition, including luminal, basal, and adipocyte populations, mimicking the mammary gland microenvironment.

The main steps in mammary organoid generation have been summarized from published protocols^114,119–123^ and enumerated below and schematically, in Fig. 2.

#### Step 1: Cell isolation and preparation

Mammary epithelial cells are isolated from breast tissue via enzymatic digestion or mechanical mincing, followed by filtration and centrifugation to obtain a clean epithelial cell population. Cells from human milk are enriched using centrifugation and carefully plated. Low cell numbers make this step challenging but manageable with optimized protocols. Reprogrammed somatic cells (iPSC) require lineage-specific differentiation into mammary epithelial cells. This involves a series of growth factors and cytokines, making the process labor-intensive and time-consuming.

#### Step 2: Embedding in extracellular matrix

All cell types are embedded in a basement membrane matrix rich in key extracellular matrix proteins like laminin, collage IV, entactin and heparin sulfate proteoglycan, which provide a biomimetic 3-D microenvironment. These components are able to form a gel at 37 °C, to support cell attachment and promoting cell polarization, and enhancing cell-cell interactions.

#### Step 3: Organoid culture and differentiation

The embedded cells are cultured in organoid-specific media supplemented with essential growth factors. For iPSCs, differentiation protocols involve multiple media changes with precise combinations of epidermal growth factor (EGF), fibroblast growth factor (FGF), and Wnt activators to induce mammary epithelial lineage commitment.

#### Step 4: Induction of lactation

Prolactin supplementation is a pivotal step for inducing lactation-specific functionality in the organoids. Prolactin, in combination with hydrocortisone and insulin, activates the lactation machinery by upregulating milk protein genes such as casein and alpha-lactalbumin. This step is essential for mimicking lactating mammary physiology in cells derived under non-lactogenic conditions, including mammary tissue and iPSC.

#### Step 5: Maturation and analysis

After 10–14 days of culture, organoids begin to exhibit alveolar-like structures and milk protein expression. Organoids derived from tissue and milk cells achieve this stage more rapidly due to their pre-differentiated state, whereas iPSC-derived organoids take significantly longer to mature.

The use of basement membrane extracts such as Cultrex^®^ or Matrigel^®^ ensures robust growth and differentiation, while prolactin supplementation is indispensable for functional maturation. These protocols underscore the relative ease of using tissue and milk-derived cells compared to the labor-intensive process required for iPSC differentiation, highlighting the trade-offs between source availability, time, and reproducibility.

## Benefits and limitations of using human milk models

**Fig. 3:**
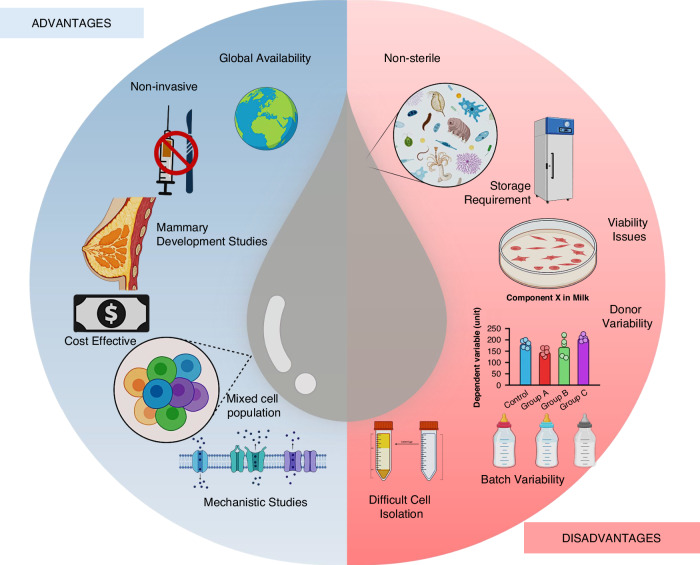
Advantages and disadvantages in human milk cell research. The schematic presents the pros and cons of harnessing human milk cells for functional and mechanistic organoid studies, weighing the ease of collection and relative affordability against challenges like propensity for microbial contamination, donor and batch variability, and labor-intensive processing and separation of lactocytes from immune cells.

Human milk has great potential for the improvement of infant nutrition and development through a better understanding of breast biology, and the development of new therapeutic solutions. Figure 3 summarizes the advantages and disadvantages associated with human milk research. Despite many advantages, we also note the challenges and limitations in working with human milk arising from the inherent heterogeneity and variability of milk cells depending on donor, lactation stage and environmental factors. The composition of human milk can be influenced by maternal variables such as nutrition, stress, and infections. For instance, mastitis infection can modify gene expression patterns in milk cells,^46^ potentially impacting function. Table 2 specifically focuses on the technical aspects and experimental considerations of utilizing milk-derived cells. For example, it lists the capability for single-cell analysis, allowing researchers to distinguish between different epithelial subpopulations. However, it notes important limitations, such as variable cell viability between donors, the potential need for tailored culture conditions, and the presence of immune cells that might complicate epithelial specific studies. The accessibility of milk makes repeat and non-invasive sampling possible, though factors like donor physiology and sample handling can lead to inconsistencies. Analyzing the influence of these maternal variables on milk cells will be essential for interpreting research findings and designing treatments to improve lactation.^124,125^

**Table 2:** Technical benefits and limitations of milk-derived epithelial cells for mammary gland research.^119,131^

Properties	Benefits	Limitations
Cell viability	Milk-derived epithelial cells are viable, contrary to historical belief	Viability may vary between donors and across lactation stages
In vitro culture	Cells can be maintained in vitro similar to breast tissue-derived cells	Culture conditions may need optimization for long-term proliferation and differentiation
Transcriptional identity	Exhibit transcriptional resemblance to luminal progenitor cells, indicating relevance for developmental studies	May not fully capture the diversity of epithelial cell types found in the entire mammary gland
Single-cell resolution	Enables identification of distinct epithelial subpopulations (e.g., two luminal secretory clusters)	Requires high-throughput and costly technologies (e.g., scRNA-seq)
Presence of immune cells	Shared immune cell clusters observed in both milk and NMCs highlight immune-mammary interactions	May complicate epithelial cell-specific analyses unless sorted
Non-invasive sampling	Easily accessible compared to other tissue biopsies, making longitudinal studies feasible	Cell yield and consistency may be affected by donor physiology and sample handling

## Conclusion and prospective future directions

Human milk offers a non-invasive and readily available window into the physiology of lactation through the culturing of mammary organoids, harvesting of milk fat globules and extracellular vesicles and isolation of bioactive components, immune and stem cells. This review demonstrates the diverse and most promising applications of human milk, from the immunotherapeutic effects of extracellular vesicles to the diagnostic use of epigenetic and genetic markers in milk. Some of these technologies, such as the manufacturing of artificial or lab-grown milk, while intriguing and potentially impactful from a sustainable and ethical viewpoint, are still in early stages of development and face significant challenges of economic scale. Still others are largely unrealized, including the harvesting of stem cells in milk for applications in regenerative medicine, such as cell replacement therapy in neurodegenerative disorders. Future collaborations between academics, clinicians and milk banks, especially in low-income countries, should be fostered for greater access to the potential applications of human milk.^126^ The prospective utilization of mammary organoids in conjunction with bioprinting technology presents considerable potential for advancing tissue engineering, disease modeling, and precision medicine.

We highlight safety concerns from the increasing burden of xenobiotics from environmental pollutants and medications that impact lactating persons, and the potential for viral spillover into milk in the face of new pandemic threats. Milk-derived extracellular vesicles have exhibited the capacity to influence gut microbiota, facilitate tissue healing, and convey regulatory signals to developing tissues, thereby emulating certain natural advantages of human milk. Integrating EVs into milk formula could enhance individualized newborn nutrition and help reconcile the differences between formula and human milk in promoting early-life gut and immunological health. We foresee a growing role for milk biobanks in supporting longitudinal research on the composition of human milk using state of the art proteomic, metabolomic and transcriptomic approaches.

In light of recent shifts in funding priorities in the United States which discourage projects relying solely on animal-based studies, there has been a growing emphasis on developing alternative model systems. Among these, human organoids have emerged as one of the most promising platforms, offering physiologically relevant, scalable, and ethically favorable models that bridge the gap between traditional cell culture and in vivo systems. While mammary organoid research is well established, organoids generated from human milk cells are underreported, pointing to promising opportunities for productive research in the future.
